# Morphometric Comparison and Prognostic Analysis of Permanent Maxillary Central Incisors with Eruption Disturbances—A Cross-Sectional Study

**DOI:** 10.3390/children11030307

**Published:** 2024-03-05

**Authors:** Yuri Jeong, Jonghyun Shin, Soyoung Park, Taesung Jeong, Eungyung Lee

**Affiliations:** 1 Department of Pediatric Dentistry, School of Dentistry, Dental and Life Science Institute, Pusan National University, Yangsan 50612, Republic of Korea; wjddbfl98@pusan.ac.kr (Y.J.); jonghyuns@pusan.ac.kr (J.S.); syparkpedo@pusan.ac.kr (S.P.); tsjeong@pusan.ac.kr (T.J.); 2 Department of Pediatric Dentistry, Dental Research Institute, Pusan National University Dental Hospital, Yangsan 50612, Republic of Korea

**Keywords:** upper central incisor, unilateral eruption disturbance, morphometry, surgical intervention

## Abstract

Aims: The aim of this study was to retrospectively compare the morphometrics of permanent maxillary central incisors with and without eruption disturbances, while simultaneously evaluating prognosis based on different factors. Materials and Methods: Seventy patients with unilateral permanent maxillary central incisor eruption disturbances were included. Within a group of 70 subjects, measurements were taken for both normally erupted central incisors and central incisors with eruption disturbances to determine the length of the roots and the volume of the teeth. Various factors, such as angulation of impaction, and vertical height of impaction, were assessed to investigate their correlation with surgical intervention. Results: Both the root length and tooth volume were significantly smaller in the eruption disturbance incisors than in the normally erupted incisors (*p* ≤ 0.001). Moreover, there was a statistically significant increase in surgical intervention among cases with no clear physical barrier (primary retention) (*p* < 0.05) or when adjacent normally erupted central incisors exhibited more than 2/3 of root development (*p* < 0.05). Conclusions: The results of this study numerically demonstrated the delayed tooth development of the permanent maxillary central incisors with unilateral eruption disturbances compared to appropriately erupted incisors by measuring root length and tooth volume. The absence of obstacles and the degree of root development in adjacent erupted incisors might serve as factors for clinicians to determine the necessity and timing of surgical intervention.

## 1. Introduction

The permanent maxillary central incisor, positioned prominently at the center of the face typically erupts 7–8 years of age during the transition from deciduous to mixed dentition [1,2,3,4,5]. Failure to erupt during this timeframe may result in aesthetic and socio-psychological concerns that could compromise oral health-related quality of life (OHRQoL) due to potential implications for social attractiveness upon initial encounter. Furthermore, it can give rise to dental complications such as root curvature or ankylosis in the affected tooth, which could potentially impact the normal eruption of adjacent teeth [6,7,8,9,10,11]. Consequently, the establishment of proper occlusion during the transition to permanent dentition may pose challenges. Hence, an accurate diagnosis and timely intervention for eruption disorders is crucial [12].

Factors causing eruption disturbances of permanent teeth can be broadly divided into systemic factors and local factors. Firstly, systemic factors include vitamin D-resistant rickets, endocrine disorders, long-term chemotherapy, oral clefts, as well as Gardner syndrome or Down syndrome. Additionally, according to the World Health Organization (WHO), there is still diverse opinions regarding the association between preterm infants, defined as birth before 37 weeks of gestation, or low birth weight, defined as less than 2500 g at birth, and eruption of permanent dentition [13,14]. Secondly, local factors include supernumerary teeth, odontoma, trauma affecting tooth germination, root dilaceration, crowding, or periapical lesions of primary incisors [15,16,17,18]. Identifying underlying causes and devising suitable treatment strategies are crucial in such cases.

Diagnosis of eruption disturbances involves clinical and radiological examinations. According to the clinical guidelines of Royal College of Surgeons of England 2022 [19], it could be considered as eruption disturbance of permanent maxillary incisors following:

- the contralateral incisor erupted more than six months previously;
- the upper central incisors remained unerupted for more than one year after the lower incisors had already erupted;
- significant deviation from the normal eruption sequence exists, such as lateral incisors erupting prior to central incisors

A normal eruption time difference of up to 4 months for the maxillary incisors and up to 12 months for the mandibular incisors is generally acceptable [20,21]. However, discrepancies beyond these periods or contralateral incisor eruptions preceding the affected one might suggest an eruption disturbance [15,20].

Radiographic examination provides solid support for diagnosing eruption disturbances. The first consideration is periapical radiographs, which allow for the localization of unerupted tooth position with parallax method [22,23]. While panoramic radiographs cannot replace intraoral periapical radiographs, they offer additional diagnostic information. Panoramic radiographs enable the comparison of height and inclination of impacted tooth [15,16,24,25], Due to these advantages, panoramic radiographs are used in conjunction with periapical radiographs for diagnosis. Cone-beam computed tomography (CBCT) provides precise 3D information on tooth position, relationship with adjacent teeth, and root abnormalities [26,27].

Treatment options about abnormally unerupted permanent maxillary incisors can be broadly categorized into four main approaches. Firstly, if there are physical obstructions such as supernumerary teeth, they need to be removed. Following this, a period of approximately 12 to 18 months may be expected for spontaneous eruption of the incisors. If inadequate eruption space is anticipated, simultaneous expansion of space for eruption can be considered. In cases where spontaneous eruption does not occur despite observation, more proactive measures such as surgical exposure and orthodontic traction should be pursued. In instances where severe root dilacerations or ankylosis occur despite these efforts, decoronation or extraction of incisors might be considered as a last resort [23,28].

Studies utilizing panoramic radiographs and CBCT have consistently investigated impacted maxillary canines [29,30,31,32] or the root morphology and impaction of the posterior teeth [33,34,35]. Previous studies have also reported morphological analyses of impacted maxillary central incisors [6,36,37,38,39]. However, most of these analyses were conducted in cases involving obstructive factors such as supernumerary teeth. Hence, the objectives of this study can be summarized as follows: (1) To morphometrically compare unilaterally unerupted permanent maxillary central incisors with normally erupted incisors by CBCT analysis. (2) To evaluate the differences in impaction height and angle depending on the presence or absence of obstacles causing impaction. (3) To analyze the association between various factors (history of trauma, tooth development stage, presence of physical obstacles) and the necessity of surgical intervention.

## 2. Materials and Methods

### 2.1. Subjects

A retrospective investigation was conducted on a total of 2970 patients who visited the Department of Pediatric Dentistry at Pusan National University Dental Hospital between 2015 and 2023, and underwent panoramic and CBCT imaging for various purpose.

The selection of participants was based on predefined inclusion and exclusion criteria. Specifically, we focused on cases of unilateral maxillary central incisor eruption disturbances. Eruption disturbance was characterized by the eruption of the contralateral central and lateral incisors, while the central incisor on the affected side failed to erupt. Cases related to systemic conditions affecting the eruption, bilateral maxillary central incisor eruption disturbances, and those lacking suitable radiographic records (panorama or CBCT) for analysis, imaging errors, or low-resolution quality were excluded from the study.

Within the selected subjects, normally erupted maxillary central incisors constituted the Normal group (N group), while those presenting with eruption disturbances were categorized into the Eruption Disturbance group (ED group). The ED group was further subdivided based on the criteria outlined in a previous study [40], which distinguished between primary retention and impaction, depending on clearly identifiable causes diagnosed at the time of examination. 

*Inclusion criteria*

- Unilateral eruption disturbance of maxillary central incisor
- Patient with both initial panoramic and CBCT images

*Exclusion criteria*

- Low quality of radiographic records
- History of previous orthodontic treatment
- Downs syndrome, cleft lip and/or palate, or other systemic factors associated with eruption disturbances

### 2.2. Electronic Medical Records

The retrospective analysis involved the examination of the electronic medical records of the selected subjects; a thorough review of panoramic and CBCT data was conducted. The investigated parameters included age, sex, side of eruption disturbance (right or left), history of trauma to the primary maxillary incisors. Furthermore, excluding patients who did not return for follow-up appointments, we confirmed the spontaneous eruption status based on clinical examinations and radiographic assessments, including periapical radiographs taken at 4–6 months intervals. Despite observation, if spontaneous eruption did not occur, surgical exposure and orthodontic traction were performed. These procedures are collectively referred to as “surgical intervention”.

### 2.3. Panoramic Radiograph

Through panoramic imaging, an analysis was conducted to assess the presence and type of physical obstructions such as odontoma, supernumerary tooth, and microdontia, as well as the stage of development (i.e., Demirjian’s stages) of the maxillary central incisors at the time of diagnosis. Additionally, the angle and height of impaction of the affected teeth were examined. 

**Figure 1:**
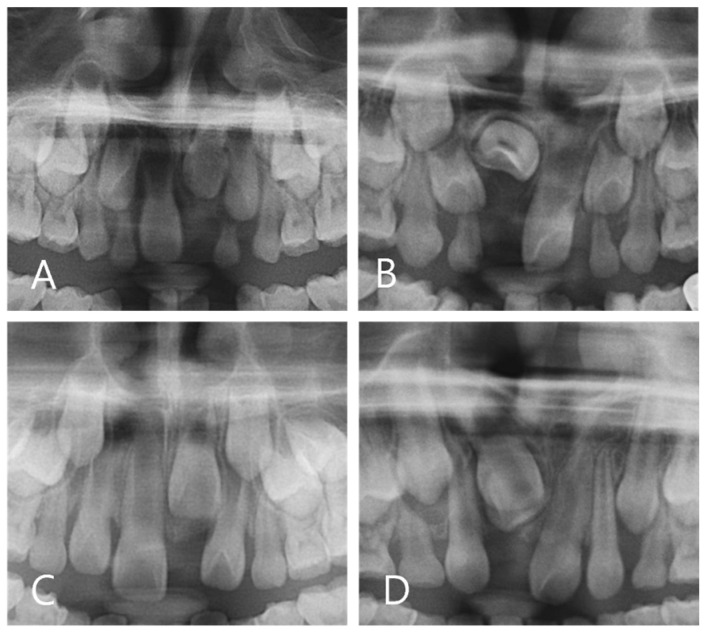
Demirjian’s stage assessment of N group. (**A**) Stage E (**B**) Stage F (**C**) Stage G (**D**) Stage H.

The Demirjian’s stages were used to classify the stages of tooth development based on panoramic images at the time of diagnosis for both the N and ED groups [6,30,41,42] (Figure 1).

**Figure 2:**
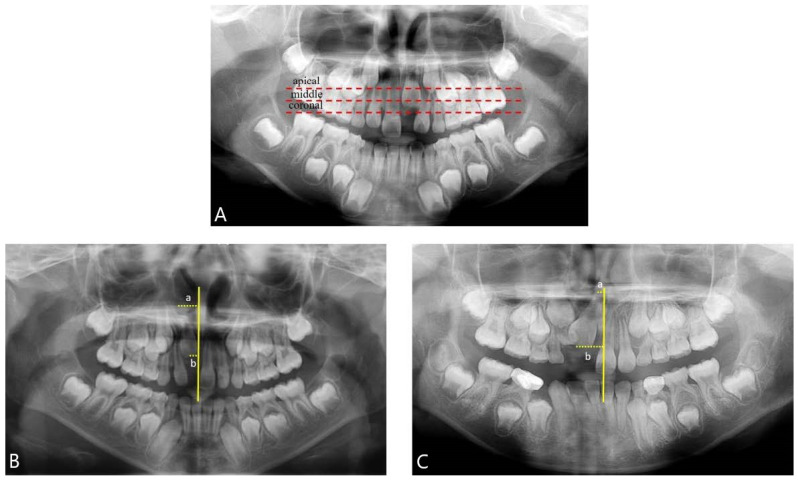
Panoramic radiographs analysis. (**A**) Criteria for evaluating impaction height; coronal, middle, and apical were classified with reference to the contralateral erupted central incisor. (**B**,**C**) Criteria for evaluating impaction angle (mesial, distal); using the vertical midline passing through the ANS (anterior nasal spine) as a reference, the distance from this line to the midpoint of the incisal edge (b) was subtracted from the distance from this line to the midpoint of the root apex (a) of the impacted central incisor. A positive value indicated a mesial impaction, while a negative value indicated a distal impaction.

The impaction angles of the affected central incisors were categorized as mesial or distal by measuring the discrepancy between the distance from the center of the apex and the center of the crown along the midline. Furthermore, the impaction height was classified into three groups (coronal, middle, and apical) according to the criteria of Smaillience et al., with reference to the contralateral erupted central incisor [12,31,43] (Figure 2).

Criteria for evaluating impaction angle (mesial, distal) involved using the vertical midline passing through the ANS (anterior nasal spine) as a reference. The distance from the line to the midpoint of the incisal edge (B) was subtracted from the distance to the midpoint of the root apex (A) of the impacted central incisor. A positive value indicated mesial impaction, whereas a negative value indicated distal impaction. 

### 2.4. CBCT Measurement

CBCT imaging for three-dimensional analysis was performed using a Viso G7 device (PLANMECA, Helsinki, Finland). The scanning parameters included voxel size of 0.3 mm, 110 kV, 11.0 mA, and a duration of 3.272 s. Subsequently, the CBCT DICOM files were subjected to three-dimensional analysis using the OnDemand 3D software (version 1.0.10.10055; Cybermed, Seoul, Republic of Korea) [6,44].

**Figure 3:**
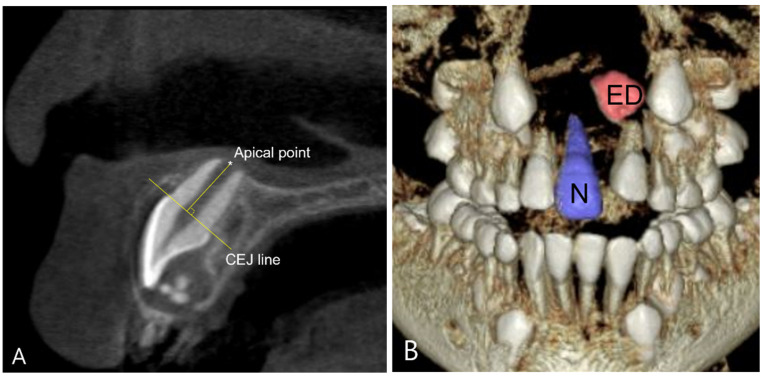
Analysis of root length and tooth volume using Cone Beam Computed Tomography (CBCT). (**A**) Measurement of root length; the shortest perpendicular distance between the line passing through the cementoenamel junction (CEJ) of the tooth and the line connecting the most apical point of the root. (**B**) Measurement of tooth volume; N group and ED group were each segmented and their volumes were measured.

Root length and tooth volume were measured for each individual in both the N and ED groups. To ensure the reliability of the test, a proficient examiner performed the measurements twice, with a one-week interval between sessions (Figure 3).

### 2.5. Statistical Analysis

All data collection and analysis were conducted by one dentist. Data were compiled using Excel and statistical analysis of all collected data was performed using SPSS program (Version 26.0. IBM Corp., Armonk, NY, USA). The significance level was set at *p* < 0.05. Data are presented as frequency with percentages for categorical variables and as mean ± standard deviation (SD) for continuous variables. Differences in characteristics among the subgroups of study participants were compared using the chi-square test for categorical variables and the independent *t*-test or Mann-Whitney U test for numerical data, as appropriate. Furthermore, differences in characteristics among subgroups of study participants were compared using analysis of variance (ANOVA) or Fisher’s exact test as deemed appropriate for the analysis.

## 3. Results

**Figure 4:**
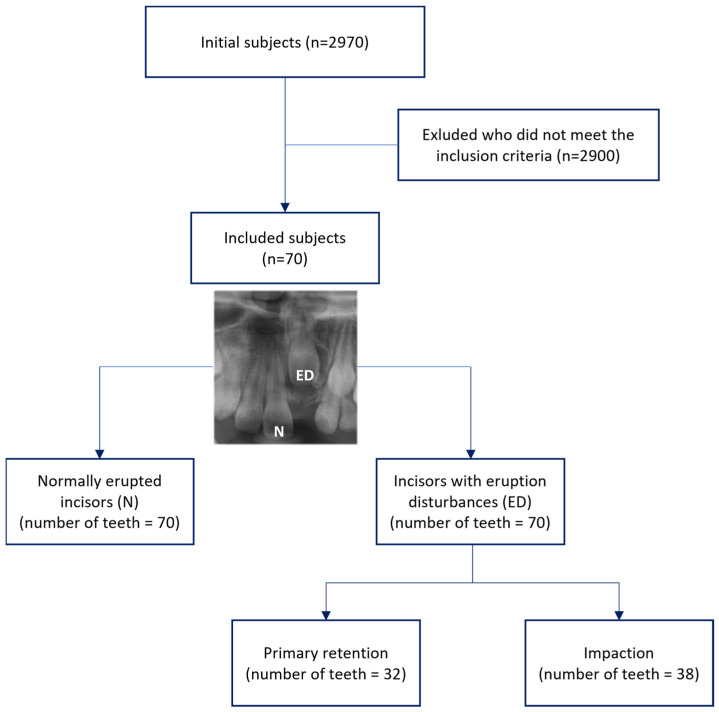
Flow chart showing process of selecting subjects included in the study.

Table 1 presents the descriptive statistics of the study cohort. From a pool of 2970 patients who underwent diagnostic panoramic and CBCT imaging at the Pediatric Dentistry Department of Pusan National University Dental Hospital between March 2015 and June 2023, a final cohort of 70 subjects meeting the research criteria was included in the study (Figure 4). This comprised 29 males (41.4%) and 41 females (58.6%), with an average age of 8.32 ± 1.27 years (range: 6.1–11.5 years) at the time of diagnosis for eruption disturbances. The distribution of the maxillary central incisors with eruption disturbances 8was 38 (54.3%) on the right and 32 (45.7%) on the left.

In the ED group, primary retention accounted for 32 cases (45.7%), whereas impaction was observed in 38 cases (54.3%), indicating a higher prevalence of impaction due to physical obstruction. Examination of trauma to the primary incisors revealed that 50 patients (71.4%) reported no history of trauma, whereas 20 patients (28.6%) had a history of trauma. Regarding impaction angles, mesial impaction was observed in 33 cases (47.1%), while distal impaction was observed in 37 cases (52.9%). In terms of the impaction height, 29 cases (41.4%) were categorized as coronal or middle, whereas 12 (17.1%) were classified as apical. The distribution of tooth development stages for the impacted central incisors in the eruption disturbance group varied from stage E to G, with E accounting for 22.9%, F for 51.4%, and G for 25.7% of cases.

The root length measured through CBCT data indicated that the N group was 9.49 ± 2.43 mm and the ED group was 6.70 ± 2.14 mm. A statistically significant difference was observed between the two groups, with the ED group showing a considerably shorter root length than the N group (*p* < 0.001).

Concerning tooth volume measurements, the N group had an average volume of 424.7 ± 90.9 mm^3^, while the ED group demonstrated a statistically significantly smaller volume with an average of 376.4 ± 90.6 mm^3^ (*p* = 0.001) (Table 2).

A study was conducted to analyze differences in root length and tooth volume according to gender and age. Although boys had larger mean root length and tooth volume compared to girls, there was no statistically significant difference (*p* > 0.05) (Table 3). When analyzed by age groups (6–7, 8–9, 10–11 years), results showed that as age increased, both root length and tooth volume exhibited larger mean values, and this difference was statistically significant (*p* < 0.05) (Table 4).

The analysis of impaction height and angle based on diagnosis indicated that there was no significant difference in impaction height between the two groups (*p* > 0.05). However, concerning the impaction angle, a noteworthy distinction was found; primary retention cases displayed a higher occurrence of mesial impaction, whereas impaction cases pre-dominantly manifested as distal impaction. This difference was statistically significant (*p* = 0.009) (Table 5).

The implementation of surgical intervention for maxillary incisors with eruption disturbances was investigated, excluding 8 subjects who did not attend follow-up observation until spontaneous eruption occurred. Among the 62 subjects, 24 (38.7%) experienced spontaneous eruption without surgical intervention, while 38 (61.3%) underwent surgical intervention. Of the 38 patients undergoing surgical treatment, 25 (61.3%) had primary retention and 13 (39.4%) showed impaction. The difference in the implementation of surgical intervention between the two groups was statistically significant (*p* = 0.001) (Table 6).

In this study, among the adjacent maxillary central incisors that erupted normally at the time of diagnosis, the Demirjian’s stages for root development were as follows: 2 cases (2.8%) were classified as stage E, 17 cases (24.3%) as stage F, 42 cases (60.0%) as stage G, and 9 cases (12.9%) as stage H. In cases of stage E, two patients underwent orthodontic traction despite the early dental age. This decision was prompted by the presence of an inverted impaction in which the crown of the affected tooth was directed upward, necessitating early intervention. The ratio of cases classified as stage F was equal between those undergoing orthodontic traction and those who did not. However, for stages G and H, the number of cases undergoing orthodontic traction was significantly higher than that of those who did not (*p* < 0.05) (Table 7).

The results of the analysis of the association between primary dentition trauma history and surgical intervention in the 62 patients (excluding 8 patients with discontinued follow-up) are as follows (Table 8). Among the 18 patients with a history of penetrating trauma, 13 (72.2%) underwent surgical intervention, while among the 44 patients without significant trauma history, surgical intervention was performed in 25 (56.8%). There was no statistically significant difference observed (*p* > 0.05).

## 4. Discussion

The prevalence of maxillary central incisor eruption disorders ranges from 0.13% to 2.0% [7,15,21], with a higher occurrence among Asians [21,45]. Maxillary central incisors that undergo normal eruptions continue to experience root growth post-eruption, with root development continuing until the ages of 7–10 years [6,46]. Therefore, early and appropriate diagnosis and intervention for eruption disorders are crucial despite their relatively low prevalence [7].

A 2014 study focusing on permanent dentition eruption disturbances reported mean ages for the diagnosis of eruption disturbances at 11.1 years for males and 10.7 years for females [47,48,49]. In the present study, the mean age of the entire cohort of 70 subjects diagnosed with unilateral maxillary central incisor eruption disorders was 8.33 ± 1.26 years. Delayed eruption of the permanent maxillary central incisors, owing to their prominent position in the facial structure, is presumed to be noticeable relatively early by patients and guardians, prompting earlier visits to the dentists.

In this study, both the root length and tooth volume of maxillary incisors with eruption disturbances were significantly smaller than those of normally erupting incisors. Specifically, the analysis divided ages into 6–7, 8–9, and 10–11 years, respectively, to compare the root length and tooth volume. In all age categories, maxillary incisors with eruption disturbances exhibited significantly smaller values compared to normally erupted incisors. This suggests that eruption disturbances may influence tooth development, underscoring the need for appropriate interventions. Furthermore, this study indicated a statistically significant difference in the impaction angles based on the presence of physical obstructions. When obstacles such as supernumerary teeth were present, there was a higher tendency for impaction towards the distal aspect, whereas in the absence of such obstacles, there was a higher tendency for impaction towards the mesial aspect. These results appear to be influenced by the occurrence location of supernumerary teeth, which induce impaction of maxillary permanent incisors.

Surgical intervention, including orthodontic traction, was initiated when clinical and radiological examinations at intervals of 4–6 months did not reveal a spontaneous eruption. In this study, we analyzed the presence of obstacles, dental age of adjacent erupted incisors, and trauma history as related factors to investigate the association with surgical intervention. The results indicated a significantly higher rate of surgical intervention in cases diagnosed with primary retention without clear obstacles causing impaction and cases where adjacent incisors had a dental age of G stage or higher, despite over one year of observation without spontaneous eruption signs. While a higher rate of surgical intervention was observed in cases with trauma history, there was no statistically significant association. Further research into the types such as avulsion or crown fracture and timing of trauma is warranted to advance this study.

The 24 cases that did not undergo surgical intervention were divided into two groups based on the treatment approach. Among these, in six cases whose analyses during mixed dentition revealed sufficient space for eruption, regular interval X-ray imaging over 4–6 months displayed spontaneous eruption. Among these cases, four exhibited hypodontia in the contralateral lateral incisor, one had a supernumerary tooth, and the remaining one had displacement of the incisor due to an apical lesion related to a traumatized tooth, which was identified as the cause of the eruption disturbance. For the remaining 18 patients with inadequate eruption space, treatment involved the use of a banded RPE or a small maxillary expander (SME) to expand the space without applying direct force to the teeth, ultimately resulting in spontaneous eruption of the impacted maxillary central incisors. 

There are several limitations to this study. Despite including a larger number of participants compared to previous studies on the morphological analysis or prognosis of maxillary central incisors with eruption disturbances [6,50], the sample size was still insufficient for detailed classification. Furthermore, while the characteristic nature of cross-sectional studies offers the advantage of comparing descriptive information, observing the longitudinal growth of incisors with eruption disturbances presents challenges. In addition, there was inherent selection bias due to the recruitment of patients from a singular geographic area, thereby limiting the generalizability of the findings to broader populations. Thus, future researches would aim to mitigate these limitations and involve more various variables in the analysis.

Consideration of radiation levels and exposure in panoramic radiographs and Cone Beam Computed Tomography (CBCT) imaging is also an important aspect. Nevertheless, the acquisition of panoramic and CBCT images offers valuable insights not only into the spatial orientation of impacted teeth in relation to adjacent structures but also into the maxillo-mandibular and dentoalveolar regions. Consequently, the diagnostic advantages conferred by these modalities may supersede the associated risks, especially in cases involving impacted teeth.

When performing surgical exposure and orthodontic traction of teeth with eruption disturbances, factors such as the risk of demineralization and bond strength need to be considered. Biomimetic materials, particularly biomimetic hydroxyapatite, are reported to be effective in reducing enamel demineralization during orthodontic treatment [51,52]. It could be interesting to evaluate enamel integrity in the pre- and post- surgical phases and maintenance using biomimetic hydroxyapatite in future research.

Previous studies have primarily focused on root development and impaction due to physical obstacles, such as supernumerary teeth, in impacted maxillary central incisors [53]. However, recent research assessing root development and associated factors in impacted maxillary central incisors, encompassing cases of primary retention without physical obstruction, has been limited. Furthermore, cases of severe tooth displacement or overlapping structures in panoramic images often pose challenges in accurately assessing root development of the affected teeth. In such instances, using root development of normally erupted central incisors as a reference point could assist clinicians in determining the optimal timing for active intervention.

## 5. Conclusions

This study, through the measurement of root length and tooth volume, revealed that permanent maxillary central incisors with eruption disturbances exhibited less growth compared to those erupted normally within the same individual. This underscores the imperative for timely intervention to facilitate normal root development.

Furthermore, based on the diagnosis classified as causes of eruption disturbances, there was no difference in impaction height, but there was a difference in impaction angle. Impaction due to obstacles such as supernumerary teeth tended to orient the crown distally, while primary retention without clear causes tended to orient the crown mesially. Lastly, the presence of obstacles and the degree of development of adjacent erupted incisors were found to be associated with the decision to perform surgical intervention. When there was no obstacle causing eruption disturbance, i.e., diagnosed as primary retention, or when the development of adjacent erupted incisors was more than 2/3 progressed, there was a higher likelihood of proactive intervention involving surgical exposure and associated orthodontic intervention.

We aimed to analyze the root development of unilaterally unerupted maxillary central incisors and determine various factors influencing the necessity of surgical intervention by clinicians. This could serve as a cornerstone for subsequent research in this domain.

## Figures and Tables

**Table 1:** Descriptive statistics of the study group (independent variables).

Characteristic	
**Age**	Year
Mean ± SD	8.32 ± 1.27
**Gender**	N (%)
Male	29 (41.4)
Female	41 (58.6)
**Side**	N (%)
Right	38 (54.3)
Left	32 (45.7)

**Table 2:** Comparison of root length and tooth volume between the N group and ED group.

	Normal (n = 70)	ED (n = 70)	*p*-Value
**Volume (mm^3^)**	424.7 ± 90.9	376.4 ± 90.6	0.001 ^1^
median (IQR)	405.3	367.6	
**Length (mm)**	9.49 ± 2.43	6.70 ± 2.14	<0.001 ^2^

Values are presented as the mean ± standard deviation. ^1^ *p* values were derived by Mann-Whitney’s U test. ^2^ *p* values were derived by independent *t*-test. Shapiro-Wilk’s test was employed for test of normality assumption.

**Table 3:** Comparison of root length and tooth volume according to gender in ED group.

	Boys (n = 29)		Girls (n = 41)		*p*-Value
	Normal	ED	Normal	ED	
**Volume (mm^3^)**	449.1 ± 106.6	404.5 ± 90.6	407.6 ± 75.5	357.3 ± 71.14	0.101
**Length (mm)**	9.95 ± 2.75	7.18 ± 2.37	9.16 ± 2.15	6.36 ± 1.93	0.182

Values are presented as the mean ± standard deviation. *p* values were derived by independent *t*-test. Shapiro-Wilk’s test was employed for test of normality assumption.

**Table 4:** Comparison of root length and tooth volume according to age in ED group.

	6–7 (n = 29)		8–9 (n = 33)		10–11 (n = 8)		*p*-Value
	Normal	ED	Normal	ED	Normal	ED	
**Volume (mm^3^)**	404.2 ± 90.9	358.4 ± 91.6	424.4 ± 74.1	377.7 ± 70.1	501.6 ± 124.1	440.3 ± 141.4	0.026
**Length (mm)**	8.22 ± 2.11	5.41 ± 1.88	10.04 ± 2.12	7.07 ± 1.30	11.79 ± 2.37	9.89 ± 2.07	<0.001

Values are presented as the mean ± standard deviation. *p* values were derived by ANOVA.

**Table 5:** Impaction height and impaction angle of the ED group.

	ED		*p*-Value
	Primary Retention	Impaction	
**Height**			
Coronal	10 (32.3)	19 (48.7)	0.279
Middle	16 (51.6)	13 (33.3)	
Apical	5 (16.1)	7 (17.9)	
**Angle**			
Mesial	20 (64.5)	13 (33.3)	0.009
Distal	11 (35.5)	26 (66.7)	

Values are frequencies with percentage in parentheses. *p* values were derived by chi-square test.

**Table 6:** Correlation between the diagnosis of ED group and the surgical intervention.

	Normal (n = 62)	ED		*p*-Value
		Primary Retention (n = 18)	Impaction (n = 44)	
**Non-surgical intervention**	24 (38.7)	4 (13.8)	20 (60.6)	<0.001
**Surgical intervention**	38 (61.3)	25 (86.2)	13 (39.4)	

Values are frequencies with percentage in parentheses. *p* values were derived by chi-square test.

**Table 7:** Correlation between the Demirjian’s dental age of N group and surgical intervention.

	Dental Age				Total	*p*-Value
	E	F	G	H		
**Non-surgical intervention**	0	8 (33.3)	16 (66.7)	0 (0.0)	24 (100.0)	0.021
**Surgical intervention**	2 (5.3)	8 (21.1)	19 (50.0)	9 (23.7)	38 (100.0)	

Values are frequencies with percentage in parentheses. *p* values were derived from Fisher’s exact test.

**Table 8:** Correlation between the trauma history and surgical intervention.

	ED		*p*-Value
	Trauma hx. (n = 18)	Non-Trauma hx. (n = 44)	
**Non-surgical intervention**	5 (27.8)	19 (43.2)	0.258
**Surgical intervention**	13 (72.2)	25 (56.8)	

Values are either frequency with percentage in parentheses. *p* values were derived by chi-square test.

## Data Availability

The data presented in this study are available upon request from the corresponding author. The data are not publicly available because they were provided in raw form to the statistician for analysis, potentially remaining incomprehensible to others without detailed explanation.
